# Postnatal Guinea Pig Brain Development, as Revealed by Magnetic Resonance and Diffusion Kurtosis Imaging

**DOI:** 10.3390/brainsci10060365

**Published:** 2020-06-12

**Authors:** Roger J. Mullins, Su Xu, Jiachen Zhuo, Steve Roys, Edna F.R. Pereira, Edson X. Albuquerque, Rao P. Gullapalli

**Affiliations:** 1 Department of Diagnostic Radiology & Nuclear Medicine, University of Maryland School of Medicine, Baltimore, MD 21201, USA; roger.mullins@nih.gov (R.J.M.); sxu@umm.edu (S.X.); jzhuo@som.umaryland.edu (J.Z.); Sroys@som.umaryland.edu (S.R.); 2 Program in Neuroscience, University of Maryland School of Medicine, Baltimore, MD 21201, USA; 3 Center for Advanced Imaging Research, University of Maryland School of Medicine (UMSOM-CAIR), Baltimore, MD 21201, USA; 4 Division of Translational Toxicology, Department of Epidemiology & Public Health, University of Maryland School of Medicine, Baltimore, MD 21201, USA; epereira@som.umaryland.edu (E.F.R.P.); Ealbuquerque@som.umaryland.edu (E.X.A.)

**Keywords:** brain, guinea pig, diffusion kurtosis imaging (DKI), brain development

## Abstract

This study used *in vivo* magnetic resonance imaging (MRI) to identify age dependent brain structural characteristics in Dunkin Hartley guinea pigs. Anatomical T_2_-weighted images, diffusion kurtosis (DKI) imaging, and T_2_ relaxometry measures were acquired from a cohort of male guinea pigs from postnatal day (PND) 18–25 (juvenile) to PND 46–51 (adolescent) and PND 118–123 (young adult). Whole-brain diffusion measures revealed the distinct effects of maturation on the microstructural complexity of the male guinea pig brain. Specifically, fractional anisotropy (FA), as well as mean, axial, and radial kurtosis in the corpus callosum, amygdala, dorsal-ventral striatum, and thalamus significantly increased from PND 18–25 to PND 118–123. Age-related alterations in DKI measures within these brain regions paralleled the overall alterations observed in the whole brain. Age-related changes in FA and kurtosis in the gray matter-dominant parietal cerebral cortex and dorsal hippocampus were less pronounced than in the other brain regions. The regional data analysis revealed that between-age changes of diffusion kurtosis metrics were more pronounced than those observed in diffusion tensor metrics. The age-related anatomical differences reported here may be important determinants of the age-dependent neurobehavior of guinea pigs in different tasks.

## 1. Introduction

The guinea pig is a translationally relevant animal model for preclinical studies of disorders that afflict the developing and the aging brain [1,2,3]. In developmental neurobiology and neurotoxicology, the guinea pig is a valuable model, because the temporal development of its brain closely resembles that of the human brain [4,5]. Likewise, placentation and hormonal control of pregnancy in humans and guinea pigs are remarkably similar [2]. In addition, levels of metabolic enzymes that inactivate a number of developmental neurotoxicants, particularly organophosphorus compounds, are more comparable between humans and guinea pigs than rats or mice [6]. In the neurobiology of aging, guinea pigs have emerged as a unique model for sporadic Alzheimer’s disease (AD), in part because of their genetic profile. Specifically, the sequence of numerous proteins known to be involved in the pathophysiology of AD, including amyloid precursor protein (APP), amyloid β (Aβ) peptides, and prenesilin 1, are highly homologous, and in some cases identical, between humans and guinea pigs [7,8]. Guinea pigs also express the truncated presenilin 2 isoform (PS2V), which is found at high levels in the brain of AD patients and has been shown to increase Aβ peptide production, as well as two alternative splicing products of the tau protein-encoding gene that modulate the formation of neurofibrillary tangles [9].

A notable disadvantage for the use of guinea pigs to model disorders of the developing or the aging brain is the limited information available regarding the structural characteristics of the guinea pig brain at different ages using modern imaging techniques. Only hand-drawn atlases and histological figures of the adult guinea pig brain are currently available [10,11,12]. While atlas-level neuroanatomic segmentation is not the major focus of the present study, we used specific modalities of *in vivo* magnetic resonance imaging (MRI), with the objective of characterizing the age-dependent structural and microstructural alterations of the brain of male guinea pigs, as they grew from juvenile age (postnatal day (PND) 18–25) to adolescence (PND 46–51) and young adulthood (PND 118–123). The specific MRI methods used in this study include diffusion kurtosis imaging (DKI), T_2_-weighted anatomical scans, and T_2_ relaxation map images. Limited T2 anatomical volumes were used: (i) to provide context for the other measures and guidance for more detailed structural MRI atlases to come and (ii) to show gross evidence of brain growth over the maturational stages.

DKI is an extension of diffusion tensor imaging (DTI) that adds a series of kurtosis measures accounting for the non-Gaussian effects of biological barriers to diffusion [13]. The added value of DKI is that it provides the standard DTI measures such as fractional anisotropy (FA), mean diffusivity (MD), axial diffusivity (AD), and radial diffusivity (RD), in addition to the corresponding measures of kurtosis such as mean kurtosis (MK), axial kurtosis (AK) and radial kurtosis (RK). The standard DTI parameters are very sensitive to white matter integrity [14,15] and are widely used in MRI research. The novel kurtosis parameters provide information regarding the complexity of the tissue microstructure based on barriers to water diffusion [16,17]. As such, DKI has shown great promise as a sensitive measure of microstructural changes in both gray and white matter [18]. Recently, it has been used successfully to detect gray-matter microstructural remodeling in mouse models of Parkinson’s disease [19,20] and acute alcohol intoxication [21], as well as in mouse [22] and rat models of traumatic brain injury [23]. According to clinical studies, DKI may also be instrumental in the diagnosis of Parkinson’s disease [24], detection of Huntington’s disease pathology [25], characterization of the pathophysiology of mild cognitive impairment/Alzheimer’s disease [26,27], and identification of microstructural changes in the developing brain [28]. A common conclusion to these studies was that DKI shows far more sensitivity to microstructural alterations in gray matter than standard DTI metrics.

T_2_ relaxation mapping enables the visualization of tissue water content, which has long been known to decrease during maturation [29,30]. The displacement of water in the brain is in turn related to the ongoing process of myelination during brain maturation. Typically, the developing brain shows an age-dependent decrease in T_2_ relaxation time, with the steepest decreases occurring during periods of myelination [31,32]. T_2_ relaxometry also serves as an adjunct measure to complement and inform the interpretation of T_2_-weighted images and diffusion kurtosis images.

This study was designed to demonstrate whether age-dependent structural characteristics of the guinea pig brain can be reliably and feasibly detected using MRI structural images, DKI, and relaxometry. To our knowledge, this is the first longitudinal in vivo brain imaging study of age-related structural characteristics of the brain of male Hartley guinea pigs at three postnatal neurodevelopmental ages.

## 2. Materials and Methods

### 2.1. Animal Model

A cohort of six male Hartley guinea pigs (Charles River Laboratories, Wilmington, MA, USA) was studied longitudinally at PND 18–25, PND 46–51, and PND 118–123. These ages were chosen to conform with known landmarks in metabolic and brain maturity. Specifically, at approximately three weeks of age (pre-adolescence), xenobiotic-metabolizing enzymes, including the carboxylesterases that inactivate organophosphorus compounds, are fully developed in guinea pigs [6]. At PND 45–70, male guinea pigs undergo puberty and experience significant hormonal changes and hippocampal remodeling, while at approximately three months of age, they are sexually mature adults [33,34,35]. The ages of individual animals were known to be within the one-week range, as indicated by the vendor. Animals were euthanized after the last (adult stage) imaging acquisition. All experiments were carried out in accordance with a protocol approved by the University of Maryland School of Medicine IACUC regarding the care and use of animals and with the principles of the 1996 Guide for the Care and Use of Laboratory Animals (#0610001).

### 2.2. In Vivo MRI

All experiments were performed on a Bruker Biospec 7.0 Tesla 30-cm horizontal bore scanner, with a BGA20S gradient system capable of producing pulse gradients of 100 mT/m in each of the three axes and interfaced to a Bruker Paravision 5.0 console. A Bruker four-element ^1^H surface coil array was used as the receiver and a Bruker 154-mm circular coil as the transmitter. Animals were anesthetized using O_2_ (1 L/min) and 2–4% isoflurane, then placed prone in an animal holder with the radiofrequency coil over the cranium. Anesthesia was maintained with a mask fitted to the face of the animals. MR methods used to track guinea pig brain maturation are described in detail below and included T_2_-weighted anatomical imaging, T_2-_relaxometry, and DKI acquisitions. These methods were chosen to track growth-related changes in volume and morphometry, brain tissue water diffusion characteristics, and microstructural integrity, respectively. The approximate total time in the scanner was 55 min for each session.

The DKI acquisition consisted of a single-shot spin-echo echo-planar imaging (EPI) sequence in the coronal plane with TR/TE_eff_ of 8500/45 ms, matrix size 96 × 96, 1-mm slice thickness, 5 images at 0 s/mm^2^ followed by two b-values at 1000 s/mm^2^ and 2000 s/mm^2^ using 30 diffusion directions, with 24 slices and 2 averages. Images were reconstructed off-line using DKI reconstruction [17]. The total imaging time was 18 min, 25 s.

T_2_-weighted coronal Magnetic Resonance (MR) images were obtained using a fast-spin echo technique with a repetition time/effective echo time (TR/TE_eff_) of 8575/43 ms, 8 echoes, 35 × 35 mm^2^ field-of-view, 256 × 256 matrix size, 1-mm slice thickness, 40 slices, and 2 averages with coverage from the olfactory bulb to the posterior cerebellum. Total imaging time was 9 min, 9 s. To obtain T_2_-relaxation maps, a multi-slice multi-echo spin echo (MSME) sequence was used to probe changes in water content indicative of tissue maturity. A lower T_2_-relaxation value indicates lower water content, and, hence, greater tissue maturity [29,31]. Four slices of the MSME spin echo images were obtained using an 8-echo read-out, where the echoes were spaced at 13 ms and ranged from 13–104 ms. The sequence used a full 180° refocusing pulse at a TR of 4s and the images were obtained at a matrix size of 256 × 256, slice thickness of 1 mm, using a single average. The four slices through the forebrain were carefully selected to enable region of interest (ROI) measurement of the T_2_-relaxation time in the amygdala, hippocampus, striatum, and thalamus. Total time was 12 min, 48 s. T2-maps were generated using an in-house developed software.

### 2.3. Data Processing

**Figure A1:**
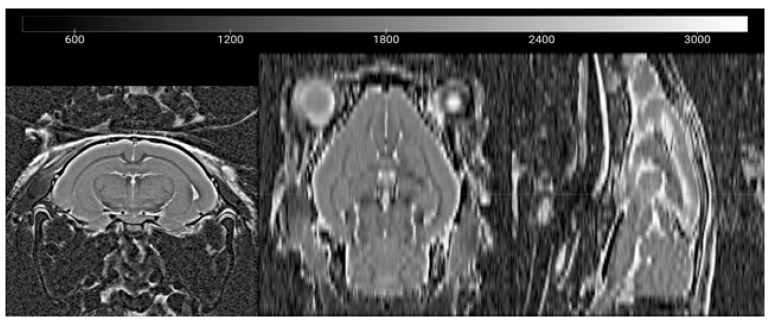
Example of T2 Volumetric Images. Representative T2-weighted image showing coronal, sagittal, and axial views of an adolescent guinea pig brain. Left–right orientation is as shown in the coronal image, the anterior (rostral) portions of the brain face upwards in the sagittal and axial views. Image created in MRIcroGL v1.2.

T_2_-weighted images were used to manually identify the regions of the forebrain after being imported to the Medical Image Processing, Analysis, and Visualization software (MIPAV v7.4.0, CIT, NIH, Bethesda, MD, USA; McAuliffe et al., 2001)[36]. Volume measures (in mm^3^) were derived by MIPAV from voxel counts within each segment and were used to calculate cerebrospinal fluid (CSF), parenchyma (tissue only), and total intracranial volume (CSF + Parenchyma). Manual skull stripping was performed on all T2-weighted guinea pig images and a guinea pig brain atlas [11] was used as a reference to aid in the identification of major anatomical landmarks. Please see Figure A1 for a representative T2 anatomical acquisition.

Images obtained from the DKI acquisition were motion-corrected and reconstructed using an in-house-developed software in MATLAB (version 7.14 R2012A, Mathworks, Natick, MA, USA), according to a previously published method [17]. This method was used to generate maps of the various diffusion parameters including FA, MD, RD, AD, RK, and MK. These parameters provide measures of the directional preference of water diffusion (FA), mean diffusion or kurtosis in all directions (MD, MK), and diffusion or kurtosis in the axial and radial directions (AD, AK, RD, RK).

**Figure 1:**
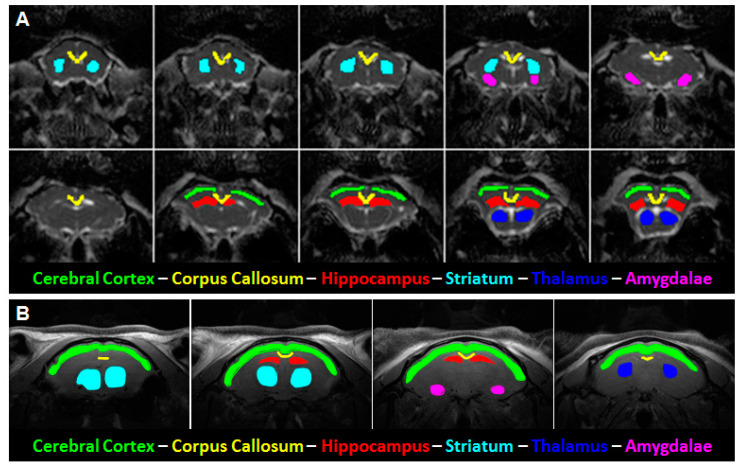
Representative mean diffusivity (MD) and T2-weighed images with delineated regions of interest (ROIs). (**A**) ROIs used for diffusion tensor imaging (DTI) and diffusion kurtosis (DKI) analyses were manually outlined on the MD image. (**B**) ROIs used for T2 relaxometry were manually outlined on a matching T2 image (left, showing cortical ROI). The slope of the ROI intensity over multiple echo times (TE) within the image was automatically plotted and fitted to arrive at the calculated T2 relaxation time.

**Figure A2:**
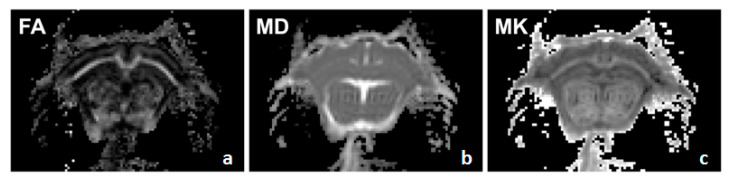
Example of DKI Contrasts. Shown are three identical coronal slices of a normal adult guinea pig brain viewed using the three main DKI parametric images including fractional anisotropy image (**a**), mean diffusivity image (**b**), and mean kurtosis image (**c**). Brighter areas of the image indicate a higher value/intensity in the indicated DKI parameter. In this medial slice, the cortex, hippocampus, and thalamus can be seen looking from top to bottom in each image. Image created in FSLview thresholded at 0 (lowest) and 1 (highest).

Regions of interest (ROIs) analyzed in diffusion images included the corpus callosum, parietal cerebral cortex, dorsal hippocampus, thalamus, dorsal and ventral striatum, and the amygdala (see Figure 1). Given the EPI distortion in the DKI images, these ROIs are representative portions of these structures rather than parcellations of the entire structure. Please see Figure A2 for the extent of image distortion using the diffusion echo-planar imaging sequence in representative slices from the FA, MD, and MK maps. Laterally non-continuous ROIs, such as the amygdala and striatum, were pooled as combined voxel means. Several consecutive slices were selected for each ROI, dependent upon their visibility in the coronal view. The corpus callosum was selected to provide a white matter reference and included 5–6 medial slices covering the area of the corpus callosum located between the cingulum bundles bilaterally. The parietal cerebral cortex and thalamus spanned over the same 2–3 slices used for the dorsal hippocampus for consistency. The striatum included 3–4 slices from its first visible rostral appearance and likewise with the 1–2 slices for the amygdala from when it was first visible. See Figure 1A for a representative example of the MD parameter with highlighted ROIs.

To measure regional T_2_-relaxation values, ROIs were manually outlined in images obtained in the first echo of the MSME sequence and extended to the rest of the multiple echo images. These ROIs included the amygdala, parietal cerebral cortex, dorsal hippocampus, striatum, and thalamus (Figure 1B). The data from each of the echoes was then fit to a single exponential T_2_-decay curve, from which the time constants were estimated.

### 2.4. Statistical Analysis

Statistical comparisons were made using the multivariate General Linear Model (GLM) Repeated Measures function in SPSS (SPSS Statistics for Windows, Version 21, IBM Corp., Armonk, NY, USA). The three chosen developmental time points (juvenile, adolescent, adult) were used as a within-subject repeated measures factor, following the six male guinea pigs over the course of maturation. The Tukey post-hoc test was used for pairwise comparisons between the developmental time points.

## 3. Results

### 3.1. Brain Growth during Postnatal Maturation

Total intracranial volume increased by approximately 15% and 11%, with maturation between the juvenile and adolescent ages and between the adolescent and young adult ages (F(2,10) = 490.64, *p* < 0.001), respectively (Table 1). Length, as measured from the anterior olfactory bulb to posterior cerebellum, increased by 15% (F(2,10) = 47.5, *p* < 0.001), and brain width between the temporal poles increased by 8% (F(2,10) = 262.04, *p* < 0.001), from juvenile to young adult ages (Table 1). The same pattern of growth is evident regarding the brain parenchymal (white and gray matter) tissue volume, which increased by about 28% from juvenile age to adulthood (F(2,10) = 611.79, *p* < 0.001). The CSF volume increased by ~50% during the same time frame (F(2,10) = 46.66, *p* < 0.001) (Table 1). The T2-weighed images from which these measures were derived had a relatively large slice thickness (1 mm). This resulted in partial volume effects that precluded the accurate parcellation of finer structures or reliable segmentation. See Figure A2 for a qualitative display of a representative T2-weighted image of the adolescent male guinea pig brain. 

Body weight increased at a much faster rate than brain volume, as expected for a precocial species (Table 1). Between the juvenile and adolescent ages and between the adolescent and young ages, the body weight of male guinea pigs increased by approximately 88% and 85%, respectively (F(2,10) = 1219.35, *p* < 0.001).

Body weight and brain volumetric measures from male guinea pigs subjected to in vivo MRI at PND 18–25 (juvenile), PND 46–51 (adolescent), and PND 118–123 (young adult). “Intracranial” volume represents the sum of the parenchyma and CSF volumes. Brain length was measured from anterior olfactory bulb tip to the posterior end of the cerebellum; brain width was measured from the far-left to the far-right temporal poles.

### 3.2. Age Dependency of Whole Brain T2, FA, Diffusivity, and Kurtosis Measures

**Figure 2:**
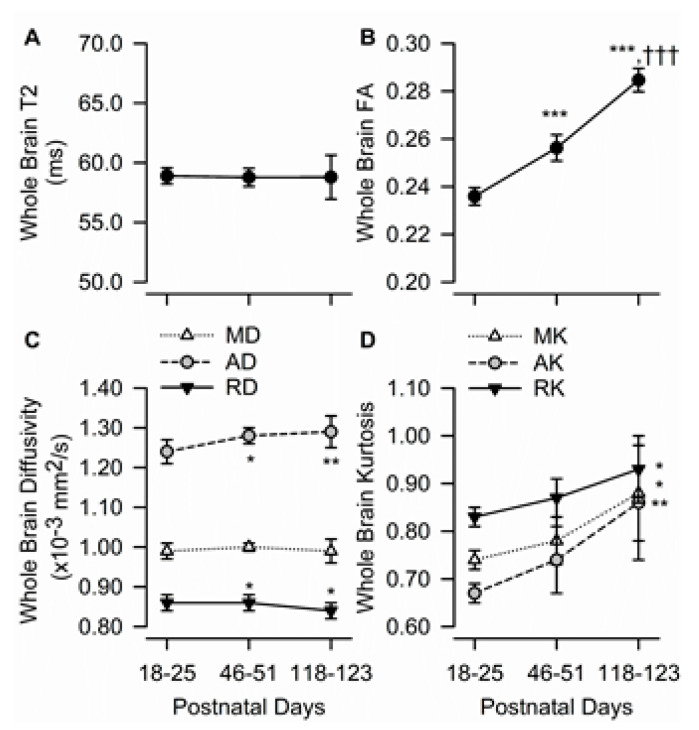
Age dependence of T_2_, fractional anisotropy, diffusivity, and kurtosis measures in the whole brain of male guinea pigs. Graphs illustrate the age dependence of T_2_ (**A**), fractional anisotropy (FA) (**B**), diffusivity measures (**C**), and kurtosis measures (**D**) in the guinea pig brain. Symbols and error bars represent mean and SD of results obtained from 6 guinea pigs examined longitudinally at postnatal day (PND) 18-25 (juvenile), PND 46-51 (adolescent), and PND 118-123 (adult). Whenever ANOVA revealed a significant effect of age on a given parameter, the Tukey post-hoc test was used for multi-group comparisons. Asterisks and daggers are used for comparison with data obtained at PND 18-25 and PND 46-51, respectively. *, *p* < 0.05, **, *p* < 0.01, ***, *p* < 0.001; †††, *p* < 0.001.

Age had no significant effect on T2 values obtained from the whole brain (Figure 2A). On the other hand, whole-brain FA increased with age (F(2,10) = 203.2, *p* < 0.001) (Figure 2B). Whole brain AD also increased significantly with age (F(2,10) = 9.27, *p* = 0.005), whereas RD decreased significantly as the animals matured (F(2,10) = 6.32, *p* < 0.017). Based on post-hoc statistical data analysis, whole brain AD increased, and RD decreased significantly from PND 18–25 to PND 46–51 and did not change significantly between PND 46–51 and PND 118–123 (Figure 2C). Whole brain MD remained constant from PND 18–25 to PND 118–123.

Age had a significant effect on whole brain MK (F(2,10) = 6.31, *p* = 0.017), AK (F(2,10) = 8.37, *p* = 0.007), and RK (F(2,10) = 4.81, *p* = 0.034). Post-hoc analyses showed that values obtained from guinea pigs at PND 18–25 and PND 118–123 differed significantly (Figure 2D).

### 3.3. Age Dependence of Regional FA

**Figure 3:**
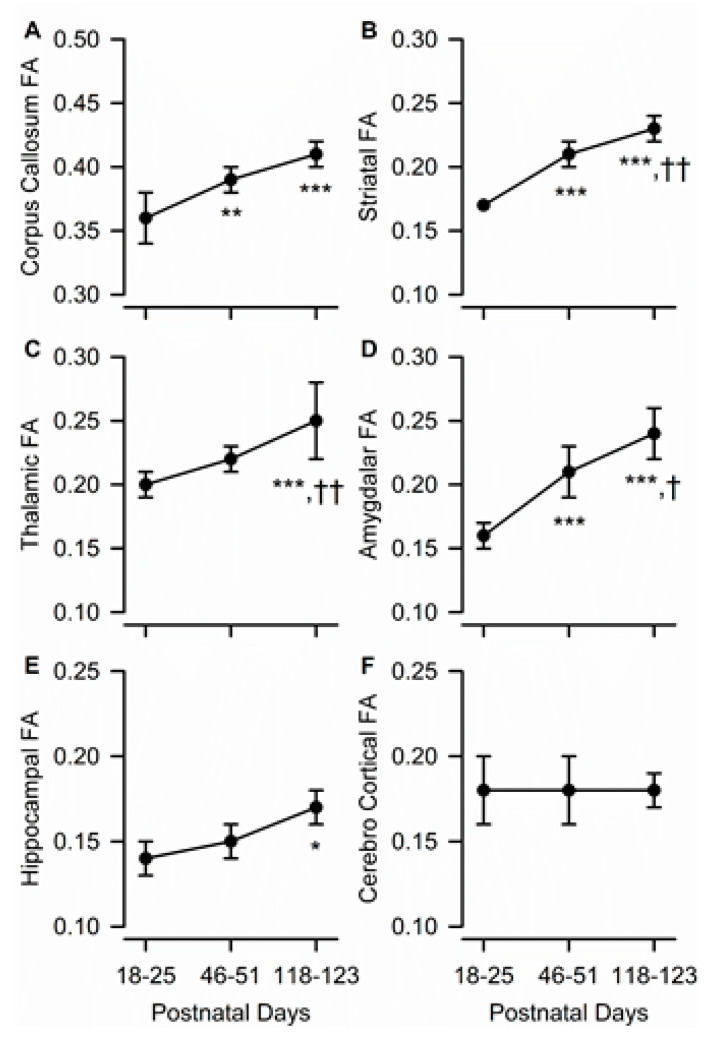
Age dependence of fractional anisotropy (FA) in different ROIs of the male guinea pig brain. Graphs illustrate the age dependence of FA in the corpus callosum (**A**), striatum (**B**), thalamus (**C**), amygdala (**D**), hippocampus (**E**), and cerebral cortex (**F**) in the brain of male guinea pigs. Symbols and error bars represent mean and SD of results obtained from six guinea pigs, examined longitudinally at PND 18-25, PND 46-51, and PND 118-123. Whenever ANOVA revealed a significant effect of age on a given parameter, the Tukey post-hoc test was used for multi-group comparisons. Asterisks and daggers are used for comparison with data obtained at PND 18-25 and PND 46-51, respectively. *, *p* < 0.05, **, *p* < 0.01, ***, *p* < 0.001; †, *p* < 0.05; ††, *p* < 0.01.

As the guinea pigs matured, FA within most ROIs increased, as it did at the whole brain level (Figure 3A–E). Specifically, age had a significant effect on FA in the amygdala (F(2,10) = 35.42, *p* < 0.001), corpus callosum (F(1.03, 5.12) = 16.26, *p* = 0.009), dorsal hippocampus (F(2,10) = 5.59, *p* = 0.023), striatum (F(2,10) = 60.48, *p* < 0.001), and thalamus (F(2,10) = 20.43, *p* < 0.001). The predominantly gray matter region of the parietal cerebral cortex was the only analyzed ROI that did not show statistically significant age-dependent changes in anisotropy (Figure 3F).

A post-hoc analysis revealed that, in the corpus callosum, age-dependent increases in FA were limited to the transition from the juvenile to the adolescent age, as FA increased significantly from PND 18–25 to PND 46–51 and did not differ statistically between PND 46–51 and PND 118–123 (Figure 3A). By contrast, in the thalamus, age-dependent increases in FA occurred during the transition from adolescence to young adulthood, with FA being comparable between PND 18–25 and PND 46–51 and significantly larger at PND 118–123 than at the younger ages (Figure 3C). In the striatum and amygdala, FA increased progressively from PND 18–25 to PND 46–51 and from PND 46–51 to PND 118–123 (Figure 3B,D). Finally, in the dorsal hippocampus, age-dependent increases in FA occurred at a slow rate, such that FA values measured at PND 118–123 were found to be significantly larger than those measured at PND 18–25 (Figure 3E).

### 3.4. Age Dependence of Regional Diffusivity Measures

**Figure 4:**
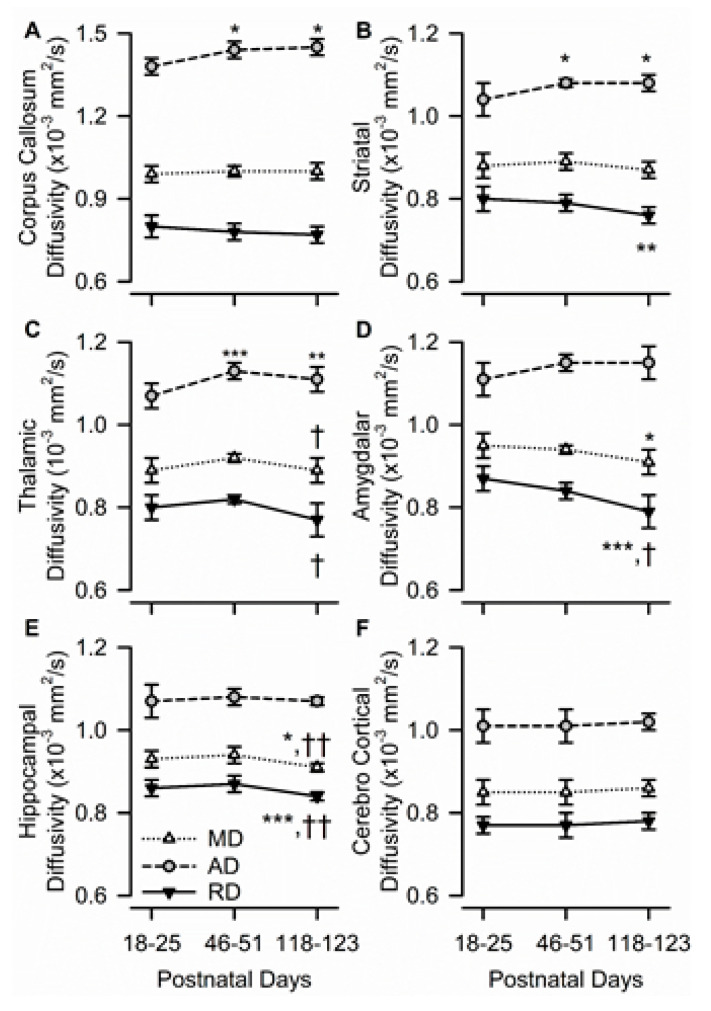
Age dependence of axial, radial, and mean diffusivity (AD, RD, and MD, respectively) in different ROIs of the male guinea pig brain. Graphs illustrate the age dependence of AD, RD, and MD in the corpus callosum (**A**), striatum (**B**), thalamus (**C**), amygdala (**D**), hippocampus (**E**), and cerebral cortex (**F**), in the brain of male guinea pigs. Symbols and error bars represent mean and SD of results obtained from six guinea pigs examined longitudinally at PND 18-25, PND 46-51, and PND 118-123. Whenever ANOVA revealed a significant effect of age on a given parameter, the Tukey post-hoc test was used for multi-group comparisons. Asterisks and daggers are used for comparison, with data obtained at PND 18-25 and PND 46-51, respectively. *, *p* < 0.05; **, *p* < 0.01; ***, *p* < 0.001; †, *p* < 0.05; ††, *p* < 0.01.

In the corpus callosum, age had a significant effect only on AD (F(2,10) = 7.35, *p* = 0.011); RD and MD remained constant with age (Figure 4A). Based on the post-hoc analysis, AD in the corpus callosum increased significantly from PND 18–25 to PND 46–51 and remained unaltered thereafter, suggesting that AD may be the main driver of FA changes.

More complex age dependencies were observed on AD, RD, and MD in the striatum and thalamus. In the striatum, age had a significant effect on AD (F(2,10) = 6.33, *p* = 0.017) and RD (F(2,10) = 8.32, *p* = 0.007), but not on MD. While striatal AD increased significantly from PND 18–25 to PND 46–51 and remained unchanged thereafter, striatal RD decreased significantly from PND 18–25 to PND 118–123 (Figure 4B). In the thalamus, age also had a significant effect on AD (F(2,10) = 14.16, *p* = 0.001) and RD (F(2,10) = 5.26, *p* = 0.027), but not on MD. While thalamic AD increased significantly from PND 18–25 to PND 46–51 and remained unchanged thereafter, thalamic RD decreased significantly from PND 46–51 to PND 118–123 (Figure 4C).

The amygdala and dorsal hippocampus shared another pattern of development, wherein age had a significant effect on MD and RD, but not on AD. In the amygdala, there were age-related decreases in both MD (F(2,10) = 4.70, *p* = 0.036) and RD (F(2,10) = 16.77, *p* < 0.001), with values measured at PND 118–123 being significantly lower than those measured at PND 18–25 (Figure 4D). Likewise, in the hippocampus, age had a significant effect on MD (F(2,10) = 10.96, *p* = 0.003) and RD (F(2,10) = 39.50, *p* < 0.001), with both values being significantly lower at PND 118–123 than at younger ages (Figure 4E). The predominantly gray matter region of the parietal cerebral cortex was the only analyzed ROI in which age had no significant effect on AD, RD, or MD (Figure 4F).

### 3.5. Age Dependence of Regional Kurtosis Measures

**Figure 5:**
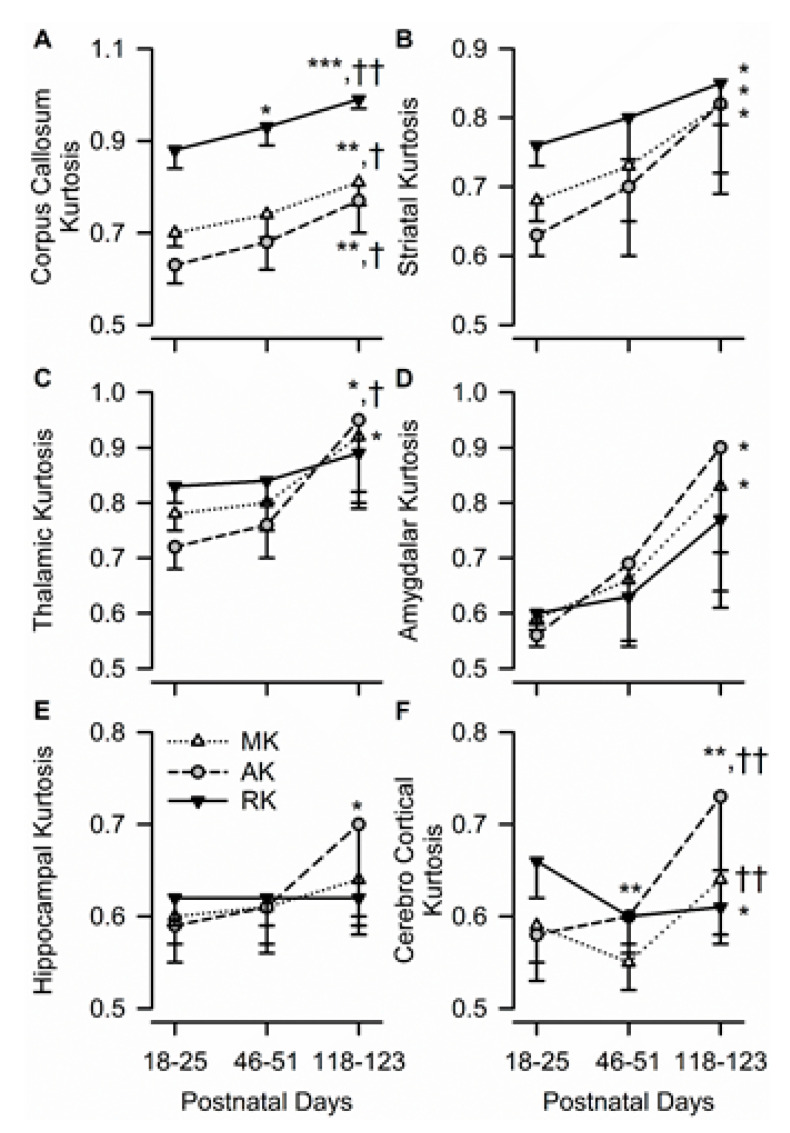
Age dependence of axial, radial, and mean kurtosis (AK, RK, and MK, respectively) in different ROIs of the male guinea pig brain. Graphs illustrate the age dependence of AK, RK, and MK in the corpus callosum (**A**), striatum (**B**), thalamus (**C**), amygdala (**D**), hippocampus (**E**), and cerebral cortex (**F**) in the brain of male guinea pigs. Symbols and error bars represent mean and SD of results obtained from six guinea pigs examined longitudinally at PND 18-25, PND 46-51, and PND 118-123. Whenever ANOVA revealed a significant effect of age on a given parameter, the Tukey post-hoc test was used for multi-group comparisons. Asterisks and daggers are used for comparison, with data obtained at PND 18-25 and PND 46-51, respectively. *, *p* < 0.05; **, *p* < 0.01; ***, *p* < 0.001; †, *p* < 0.05; ††, *p* < 0.01.

Diffusion kurtosis measures in all studied ROIs were consistently sensitive to age in the male guinea pigs. In the corpus callosum, age had a significant effect on MK (F(2,10) = 12.48, *p* = 0.002), AK (F(2,10) = 9.35, *p* = 0.005), and RK (F(2,10) = 22.04, *p* < 0.001), with all three values being significantly larger at PND 118–123 than at each of the two younger ages (Figure 5A). In the striatum, age also had a significant effect on MK (F(2,10) = 4.91, *p* = 0.033), AK (F(2,10) = 5.44, *p* = 0.025), and RK (F(2,10) = 4.75, *p* = 0.035), with all three values being larger at PND 118–123 than at PND 18–15 (Figure 5B). Age had a significant effect on thalamic MK (F(2,10) = 4.69, *p* = 0.037), thalamic AK (F(2,10) = 7.23, *p* = 0.011), amygdalar MK (F(2,10) = 4.93, *p* = 0.032), and amygdalar AK (F(2,10) = 8.29, *p* = 0.008), with all values being significantly larger at PND 118–123 than at PND 18–25 for each ROI (Figure 5C,D). In the dorsal hippocampus, age only had a significant effect on AK (F(2,10) = 4.54, *p* = 0.039), with hippocampal AK increasing significantly from PND 18–25 to PND 118–123 (Figure 5E).

The pattern of temporal development of the parietal cerebral cortex was unique compared to that of the other brain regions. Age had a significant effect on AK (F(2,10) = 14.14, *p* = 0.001), RK (F(2,10) = 9.04, *p* = 0.006), and MK (F(2,10) = 8.21, *p* = 0.008). However, while AK measured at PND 118–123 was significantly larger than that measured at younger ages and MK measured at PND 118–123 was significantly larger than that measured at PND 46–51, RK decreased significantly from PND 18–25 to PND 46–51 and remained unchanged thereafter (Figure 5F).

### 3.6. Age Dependence of Regional T_2_ Relaxation Time

**Figure 6:**
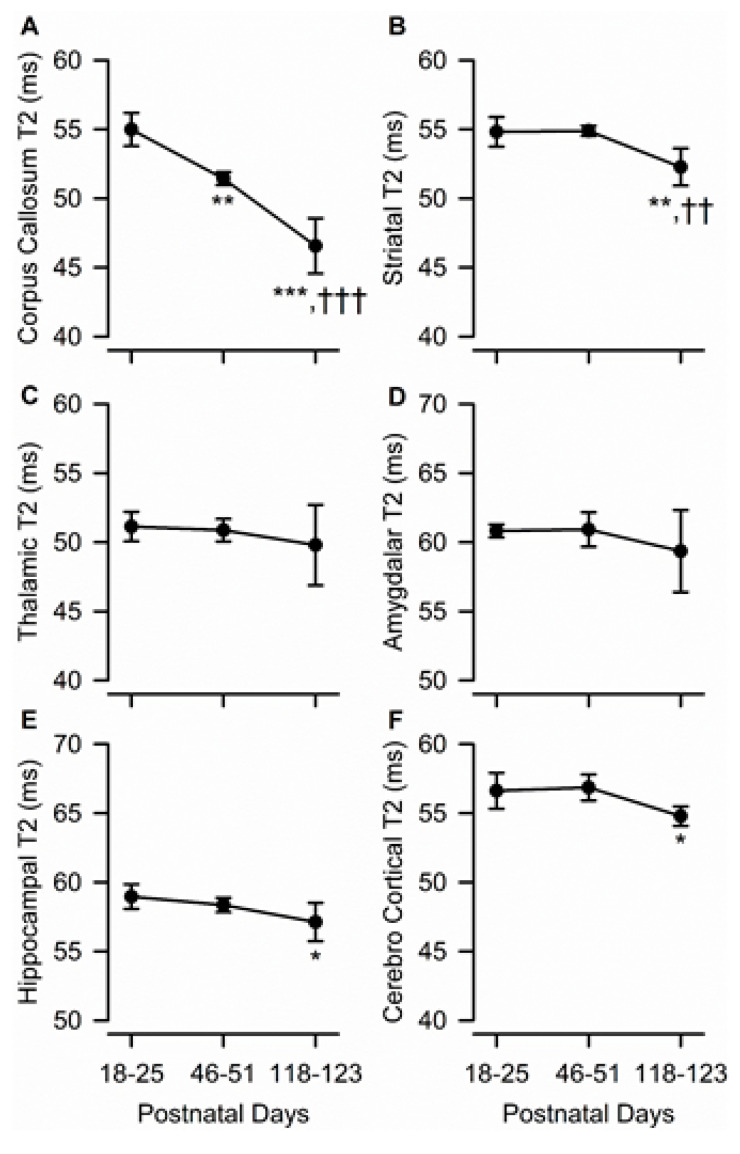
Age dependence of T2 measures obtained from different ROIs of the male guinea pig brain. Graphs illustrate the age dependence of T_2_ measures obtained from the corpus callosum (**A**), striatum (**B**), thalamus (**C**), amygdala (**D**), hippocampus (**E**), and cerebral cortex (**F**) in the brain of male guinea pigs. Symbols and error bars represent mean and SD of results obtained from six guinea pigs examined longitudinally at PND 18-25, PND 46-51, and PND 118-123. Whenever ANOVA revealed a significant effect of age on a given parameter, the Tukey post-hoc test was used for multi-group comparisons. Asterisks and daggers are used for comparison, with data obtained at PND 18-25 and PND 46-51, respectively. *, *p* < 0.05, **, *p* < 0.01, ***, *p* < 0.001; ††, *p* < 0.01; †††, *p* < 0.001.

In predominantly white matter-enriched ROIs, age had a significant effect on T_2_ relaxation time. Specifically, a significant age-dependent reduction in T_2_ values was observed in the corpus callosum (F(2,10) = 67.62, *p* < 0.001) (Figure 6A). A post-hoc multi-group comparison indicated that T_2_ values obtained from the corpus callosum of adolescent and young adult male guinea pigs were different from each other and from juvenile animals, with values decreasing by nearly 20% from PND 18–25 to PND 118–123 (Figure 6A). Smaller, albeit statistically significant, age-dependent reductions in T_2_ values were also observed in the striatum (F(2,10) = 15.95, *p* < 0.001), dorsal hippocampus (F(2,10) = 5.94, *p* = 0.020), and parietal cerebral cortex (F(2,10) = 7.52, *p* = 0.010) of male guinea pigs. Post-hoc statistical analysis indicated that, in these three ROIs, while T_2_ values were comparable between juvenile and adolescent ages, they were significantly lower at PND 118–123 than at PND 18–25 and/or PND 46–51 (Figure 6B,E,F). T_2_ values from the thalamus or amygdala did not vary significantly with age (Figure 6C,D).

## 4. Discussion

This study is the first to use T2 relaxometry, diffusivity, and kurtosis measures to characterize the postnatal maturation of cortical and subcortical structures in a cohort of male guinea pigs. Data presented here demonstrate that, as male guinea pigs mature from juvenile ages to young adulthood, the brain grows primarily in the anterior-posterior (AP) axis. In the whole brain and in different cortical and subcortical regions, especially the dorsal hippocampus and the striatum, there are age-related increases in gaussian water diffusion along the axial direction that are accompanied by reductions in water diffusion along the radial axis. There are also age-dependent increases in non-Gaussian diffusion/kurtosis along both the axial and radial directions in most brain regions, except in the parietal cerebral cortex, where non-Gaussian/kurtotic water diffusion along the radial axis decreases from the juvenile to the adolescent ages. Possible structural characteristics that underlie these findings are discussed hereafter.

In agreement with prior histological studies [5,37], the longitudinal volumetric analysis presented here indicates that, during maturation from the juvenile to young adult ages, the guinea pig brain grows primarily in the AP direction. This brain growth pattern has also been noted in an albino mouse model, wherein age-related growth occurs primarily in the AP axis, not in the other axes [38]. It remains to be determined whether this is merely a function of cranial bone growth and/or encoded in the actual maturation of the brain. Of interest, brain growth during postnatal development in humans is more symmetrical, with little deviation in overall length or shape compared to the other axes [4,39].

DKI methods applied to the whole brain provided unique insights regarding the postnatal maturation of the guinea pig brain. First, they revealed an age-dependent increase in whole brain FA and AD, accompanied by an age-dependent decrease in RD. These findings could be explained by the flow of periaxonal cytoplasm becoming more limited to a preferred direction as axons elongate and myelin integrity increases with the maturation of the animals from juvenile to young adult ages. Similar results have been reported in studies of the brain of other rodent species and of humans [28,40,41,42,43]. Second, they also revealed that whole brain MK, AK, and RK were significantly larger at young adult ages than at the juvenile and adolescence ages. Previous studies have suggested that an increase in kurtosis values is reflective of an increase in microstructural tissue complexity [13,28,44].

Among the ROIs examined, the corpus callosum, amygdala, striatum, and thalamus showed the strongest age-related increases in gaussian water movement (as measured by FA), especially along the axial direction (as measured by AD), in addition to the most pronounced age-dependent increases in non-gaussian water movement (as measured by AK, RK, and MK). By contrast, FA and AK increased only slightly, while AD, RK, and MK remained unchanged as the guinea pig dorsal hippocampus matured from the juvenile age to adulthood. In these five ROIs, RD decreased significantly between juvenile and young adult ages. Altogether, these results indicate that FA, as well as diffusivity and kurtosis measures, are sensitive to the age-dependent maturation of white matter-predominant brain regions such as the corpus callosum and gray matter-enriched regions, such as the striatum, thalamus, amygdala, and dorsal hippocampus, that have a large fraction of linear axonal tracts.

In the guinea pig parietal cerebral cortex, on the other hand, FA and AD were insensitive to the postnatal age of the animals. This is consistent with previous studies of the rat and human brain, wherein diffusivity measures remained unchanged in the cerebral cortex during postnatal neurodevelopment [41,42]. In contrast, RK decreased significantly in the parietal cerebral cortex, as guinea pigs matured from the juvenile to the adolescent age and remained unchanged thereafter, while AK increased significantly from the juvenile age to young adulthood. Thus, compared to diffusivity measures, kurtosis measures appear to more accurately resolve the age-dependent maturation of isotropic diffusion barriers in gray matter-enriched areas that, like the parietal cerebral cortex, have large numbers of crossing axonal fibers.

The age-related increase in parietal cerebral cortical AK could be partly explained by the age-dependent increased complexity of intracellular membranes and organelles in the guinea pig cerebral cortex. In fact, an earlier study reported that the height and width of pre- and postsynaptic specializations within the cerebral cortex of guinea pigs increased from late fetal ages to PND 14 [45]. On the other hand, the age-related reduction of RK is consistent with the following processes also known to take place during brain maturation: (i) transition of radial glial cells to mature astrocytes, (ii) increased axonal myelination, and/or (iii) increased neuronal dendritic complexity [46]. This is supported by a number of earlier histological findings. Specifically, the ratio of satellite perineuronal glia to neuronal cells in the guinea pig brain has been reported to increase significantly from 0.88 at 1 month of age to 1.24 at 62 months of age [47]. Likewise, myelination in the guinea pig brain reportedly increases with postnatal age at least up to PND 40 [5], and the diameter of the head and neck of dendritic spines of cerebral cortical neurons increases in guinea pigs from newborn to adult ages [48].

In gray matter- and in white matter-enriched ROIs, particularly the corpus callosum, parietal cortex, dorsal hippocampus, and striatum, T_2_-relaxation time decreased significantly as the guinea pigs matured from juvenile to young adult ages. The amount of water in the different brain regions and its interaction with the surrounding microenvironment are known to influence T_2_ [49], with conditions that restrict water diffusion leading to the reduction of T_2_ values. As such, T_2_ reductions could be explained in part by increasing myelination and cellular compaction in many ROIs during postnatal brain maturation [29,50]. However, the previously reported age-dependent decrease in water content of the brain of guinea pigs [30,32] is likely to be an equally important determinant of the age-related reduction of T_2_ during the postnatal maturation of different ROIs in the male guinea pig brain. Future histological analyses are needed to identify the exact processes that contribute to the age-dependent T_2_ reductions reported here.

The present study has some important limitations. First, the EPI distortion in the DKI images required the use of an ROI-based analysis, thus precluding the analysis of contrast maps. In future studies, issues with EPI distortion may be addressed with pre-processing steps including blip-up/blip down and diffeomorphic registration to structural images [51]. Second, the wide (1-mm) slice thickness, which was necessary to minimize the time that animals spent under anesthesia and in the MR scanner, precluded the segmentation and detailed regional volumetric analysis. Third, the longitudinal within-subject study design precluded histological verification of the anatomical differences across ages. In future studies, a standalone *in vivo* imaging session or post-mortem acquisitions at 7T at each age could yield excellent resolution and contrast suitable for generating high-resolution atlases, as well as provide the opportunity for the histological verification of age-specific anatomical characteristics. Fourth, despite the precise measurements and narrow standard deviations shown in this study, the small sample size still calls for further validation. In addition, the study was limited to the analysis of the developing male guinea pig brain; an analysis of the developing female guinea pig brain is warranted. Finally, we cannot rule out the possibility of potential developmental confounds related to isoflurane administration at each MR acquisition [52,53]. However, the previous histological findings discussed above agree with the age-dependent imaging measures obtained in the present study. Despite these limitations, the present study does provide new information regarding the guinea pig brain development that can be useful to guide future studies.

Numerous studies have reported the unique value of MRI, DTI, and DKI measures as biomarkers of the progression and severity of cognitive dysfunctions in pathological conditions, including AD, mild cognitive impairment, and organophosphorus-induced developmental neurotoxicity [17,54,55]. For example, most DKI parameters were found to be significantly reduced in gray and white matter-enriched brain regions of patients suffering from AD compared to age-matched controls and to correlate with the severity of the cognitive deficits presented by those patients [26]. Likewise, volume and cortical thickness of different brain regions were found to be reduced among children prenatally exposed to the organophosphorus insecticide chlorpyrifos, and to correlate with the decreased global intellectual quotient of these children [54]. Considering the translational relevance of the guinea pig in preclinical studies of disorders that afflict development and the adult brain [1,2,3], the present characterization of the age dependence of T_2_, DTI, and DKI measures lays the groundwork for future studies aimed at identifying neuroimaging biomarkers of the onset and progression of these pathological conditions.

In summary, analysis of T2- and diffusion-weighed images revealed that whole brain volume, gray and white matter integrity, and microstructural complexity increase to various extents in different ROIs, as the guinea pig brain matures postnatally. In white matter-rich ROIs and in gray matter-enriched ROIs containing a sizable fraction of longitudinal white matter tracts, FA and diffusivity measures were very sensitive to age. In contrast, in gray matter-rich ROIs that have a large fraction of crossing axons, T_2_ relaxometry and kurtosis measures were more sensitive to age.

## Figures and Tables

**Table 1:** Summary of Body Weight and Whole Brain Volumetrics.

Measures	Juvenile	Adolescent	Adult	
	Mean ± SD	Mean ± SD	Mean ± SD	ANOVA *p*
**Body Weight (g)**	230.9 ± 26.5	435.3 ± 25.3	806.9 ± 36.9	**<0.001**
**Volume (mm^3^)**				
**Intracranial**	3512.4 ± 164.0	4045.8 ± 163.8	4530.8 ± 133.5	**<0.001**
**Parenchyma**	3347.7 ± 153.6	3829.7 ± 159.1	4282.9 ± 141.4	**<0.001**
**Cerebrospinal Fluid (CSF)**	164.7 ± 31.7	216.1 ± 28.8	247.9 ± 17.2	**<0.001**
**Brain Length (mm)**	33.5 ± 1.2	36.5 ± 0.5	38.5 ± 0.8	**<0.001**
**Brain Width (mm)**	20.4 ± 0.4	21.4 ± 0.3	22.1 ± 0.4	**<0.001**

## References

[1] Burke R.D., Todd S.W., Lumsden E., Mullins R.J., Mamczarz J., Fawcett W.P., Gullapalli R.P., Randall W.R., Pereira E.F.R., Albuquerque E.X. (2017). Developmental neurotoxicity of the organophosphorus insecticide chlorpyrifos: From clinical findings to preclinical models and potential mechanisms. J. Neurochem..

[2] Morrison J.L., Botting K.J., Darby J.R.T., David A.L., Dyson R.M., Gatford K.L., Gray C., Herrera E.A., Hirst J.J., Kim B. (2018). Guinea pig models for translation of the developmental origins of health and disease hypothesis into the clinic. J. Physiol..

[3] Salazar C., Valdivia G., Ardiles Á.O., Ewer J., Palacios A.G. (2016). Genetic variants associated with neurodegenerative Alzheimer disease in natural models. Biol. Res..

[4] Dobbing J., Sands J. (1973). Quantitative growth and development of human brain. Arch. Dis. Child..

[5] Dobbing J., Sands J. (1970). Growth and development of the brain and spinal cord of the guinea pig. Brain Res..

[6] Ecobichon D.J., Dykeman R.W., Hansell M.M. (1978). The development of hepatic drug-metabolizing enzymes in perinatal guinea pigs: A biochemical and morphological study. Can. J. Biochem..

[7] Beck M., Müller D., Bigl V. (1997). Amyloid precursor protein in guinea pigs--complete cDNA sequence and alternative splicing. Biochim. Biophys. Acta.

[8] Johnstone E.M., Chaney M.O., Norris F.H., Pascual R., Little S.P. (1991). Conservation of the sequence of the Alzheimer’s disease amyloid peptide in dog, polar bear and five other mammals by cross-species polymerase chain reaction analysis. Brain Res. Mol. Brain Res..

[9] Sharman M.J., Moussavi Nik S.H., Chen M.M., Ong D., Wijaya L., Laws S.M., Taddei K., Newman M., Lardelli M., Martins R.N. (2013). The Guinea Pig as a Model for Sporadic Alzheimer’s Disease (AD): The Impact of Cholesterol Intake on Expression of AD-Related Genes. PLoS ONE.

[10] Potter G., Brueck W. (1958). Nervous System of Guinea Pig. Bios.

[11] Rapisarda C., Bacchelli B. (1977). The brain of the guinea pig in stereotaxic coordinates. Arch. Sci. Biol. (Bologna).

[12] Tindal J.S. (1965). The forebrain of the guinea pig in stereotaxic coordinates. J. Comp. Neurol..

[13] Cheung M.M., Hui E.S., Chan K.C., Helpern J.A., Qi L., Wu E.X. (2009). Does diffusion kurtosis imaging lead to better neural tissue characterization? A rodent brain maturation study. Neuroimage.

[14] Basser P.J. (1995). Inferring microstructural features and the physiological state of tissues from diffusion-weighted images. NMR Biomed..

[15] Saunders M., Magnanti B.L., Correia Carreira S., Yang A., Alamo-Hernández U., Riojas-Rodriguez H., Calamandrei G., Koppe J.G., Krayer von Krauss M., Keune H. (2012). Chlorpyrifos and neurodevelopmental effects: A literature review and expert elicitation on research and policy. Environ. Health.

[16] Hui E.S., Cheung M.M., Qi L., Wu E.X. (2008). Towards better MR characterization of neural tissues using directional diffusion kurtosis analysis. Neuroimage.

[17] Zhuo J., Xu S., Proctor J.L., Mullins R.J., Simon J.Z., Fiskum G., Gullapalli R.P. (2012). Diffusion kurtosis as an in vivo imaging marker for reactive astrogliosis in traumatic brain injury. Neuroimage.

[18] Xu S., Zhuo J., Racz J., Shi D., Roys S., Fiskum G., Gullapalli R. (2011). Early microstructural and metabolic changes following controlled cortical impact injury in rat: A magnetic resonance imaging and spectroscopy study. J. Neurotrauma.

[19] Arab A., Ruda-Kucerova J., Minsterova A., Drazanova E., Szabó N., Starcuk Z., Rektorova I., Khairnar A. (2019). Diffusion Kurtosis Imaging Detects Microstructural Changes in a Methamphetamine-Induced Mouse Model of Parkinson’s Disease. Neurotox. Res..

[20] Khairnar A., Ruda-Kucerova J., Szabó N., Drazanova E., Arab A., Hutter-Paier B., Neddens J., Latta P., Starcuk Z., Rektorova I. (2017). Early and progressive microstructural brain changes in mice overexpressing human α-Synuclein detected by diffusion kurtosis imaging. Brain Behav. Immun..

[21] Chen X., Zeng J., Shen Z.-W., Kong L., Zheng W. (2017). Diffusion Kurtosis Imaging Detects Microstructural Changes in the Brain after Acute Alcohol Intoxication in Rats. Biomed Res. Int..

[22] Yu F., Shukla D.K., Armstrong R.C., Marion C.M., Radomski K.L., Selwyn R.G., Dardzinski B.J. (2017). Repetitive Model of Mild Traumatic Brain Injury Produces Cortical Abnormalities Detectable by Magnetic Resonance Diffusion Imaging, Histopathology, and Behavior. J. Neurotrauma.

[23] Wang M.-L., Yu M.-M., Yang D.-X., Liu Y.-L., Wei X.-E., Li W.-B. (2018). Longitudinal Microstructural Changes in Traumatic Brain Injury in Rats: A Diffusional Kurtosis Imaging, Histology, and Behavior Study. Am. J. Neuroradiol..

[24] Wang J.-J., Lin W.-Y., Lu C.-S., Weng Y.-H., Ng S.-H., Wang C.-H., Liu H.-L., Hsieh R.-H., Wan Y.-L., Wai Y.-Y. (2011). Parkinson Disease: Diagnostic Utility of Diffusion Kurtosis Imaging. Radiology.

[25] Blockx I., Verhoye M., Van Audekerke J., Bergwerf I., Kane J.X., Delgado y Palacios R., Veraart J., Jeurissen B., Raber K., von Hörsten S. (2012). Identification and characterization of Huntington related pathology: An in vivo DKI imaging study. Neuroimage.

[26] Gong N.-J., Wong C.-S., Chan C.-C., Leung L.-M., Chu Y.-C. (2013). Correlations between microstructural alterations and severity of cognitive deficiency in Alzheimer’s disease and mild cognitive impairment: A diffusional kurtosis imaging study. Magn. Reson. Imaging.

[27] Xue Y., Zhang Z., Wen C., Liu H., Wang S., Li J., Zhuge Q., Chen W., Ye Q. (2019). Characterization of Alzheimer’s Disease Using Ultra-high b-values Apparent Diffusion Coefficient and Diffusion Kurtosis Imaging. Aging Dis..

[28] Paydar A., Fieremans E., Nwankwo J.I., Lazar M., Sheth H.D., Adisetiyo V., Helpern J.A., Jensen J.H., Milla S.S. (2014). Diffusional Kurtosis Imaging of the Developing Brain. Am. J. Neuroradiol..

[29] Ferrie J.C., Barantin L., Saliba E., Akoka S., Tranquart F., Sirinelli D., Pourcelot L. (1999). MR assessment of the brain maturation during the perinatal period: Quantitative T2 MR study in premature newborns. Magn. Reson. Imaging.

[30] Wender M., Hierowski M. (1960). The Concentration of electrolytes in the developing nervous system with special reference to the period of myelination. J. Neurochem..

[31] Holland B.A., Haas D.K., Norman D., Brant-Zawadzki M., Newton T.H. (1986). MRI of normal brain maturation. AJNR. Am. J. Neuroradiol..

[32] Leppert I.R., Almli C.R., McKinstry R.C., Mulkern R.V., Pierpaoli C., Rivkin M.J., Pike G.B. (2009). T(2) relaxometry of normal pediatric brain development. J. Magn. Reson. Imaging.

[33] Bartesaghi R., Guidi S., Severi S., Contestabile A., Ciani E. (2003). Sex differences in the hippocampal dentate gyrus of the guinea-pig before puberty. Neuroscience.

[34] Wolfer D.P., Lipp H.P. (1995). Evidence for physiological growth of hippocampal mossy fiber collaterals in the guinea pig during puberty and adulthood. Hippocampus.

[35] Hennessy M.B., Hornschuh G., Kaiser S., Sachser N. (2006). Cortisol responses and social buffering: A study throughout the life span. Horm. Behav..

[36] McAuliffe M.J., Lalonde F.M., McGarry D., Gandler W., Csaky K., Trus B.L. Medical Image Processing, Analysis and Visualization in clinical research. Proceedings of the 14th IEEE Symposium on Computer-Based Medical Systems, CBMS.

[37] Altman J., Das G.D. (1967). Postnatal neurogenesis in the guinea-pig. Nature.

[38] Slotnick B.M., Leonard C.M. (1981). Stereotaxic atlas of the albino mouse forebrain. Ann. Neurol..

[39] Pfefferbaum A., Mathalon D.H., Sullivan E.V., Rawles J.M., Zipursky R.B., Lim K.O. (1994). A quantitative magnetic resonance imaging study of changes in brain morphology from infancy to late adulthood. Arch. Neurol..

[40] Bockhorst K.H., Narayana P.A., Liu R., Ahobila-Vijjula P., Ramu J., Kamel M., Wosik J., Bockhorst T., Hahn K., Hasan K.M. (2008). Early postnatal development of rat brain: In vivo diffusion tensor imaging. J. Neurosci. Res..

[41] Calabrese E., Johnson G.A. (2013). Diffusion tensor magnetic resonance histology reveals microstructural changes in the developing rat brain. Neuroimage.

[42] Hüppi P.S., Dubois J. (2006). Diffusion tensor imaging of brain development. Semin. Fetal Neonatal Med..

[43] Larvaron P., Bielicki G., Boespflug-Tanguy O., Renou J.-P. (2006). Proton MRS of early post-natal mouse brain modifications in vivo. NMR Biomed..

[44] Mukherjee P., Miller J.H., Shimony J.S., Philip J.V., Nehra D., Snyder A.Z., Conturo T.E., Neil J.J., McKinstry R.C. (2002). Diffusion-tensor MR imaging of gray and white matter development during normal human brain maturation. AJNR Am. J. Neuroradiol..

[45] Jones D.G., Dittmer M.M., Reading L.C. (1974). Synaptogenesis in guinea-pig cerebral cortex: A glutaral-dehyde-PTA study. Brain Res..

[46] Steven A.J., Zhuo J., Melhem E.R. (2014). Diffusion kurtosis imaging: An emerging technique for evaluating the microstructural environment of the brain. AJR Am. J. Roentgenol..

[47] Brownson R.H. (1960). The effect of x-irradiation on the perineuronal satellite cells in the cortex of aging brains. J. Neuropathol. Exp. Neurol..

[48] Schüz A. (1986). Comparison between the dimensions of dendritic spines in the cerebral cortex of newborn and adult guinea pigs. J. Comp. Neurol..

[49] Mathur-De Vré R. (1984). Biomedical implications of the relaxation behaviour of water related to NMR imaging. Br. J. Radiol..

[50] Masumura M. (1987). Proton relaxation time of immature brain. II. In vivo measurement of proton relaxation time (T1 and T2) in pediatric brain by MRI. Childs Nerv. Syst..

[51] Irfanoglu M.O., Modi P., Nayak A., Hutchinson E.B., Sarlls J., Pierpaoli C. (2015). DR-BUDDI (Diffeomorphic Registration for Blip-Up blip-Down Diffusion Imaging) method for correcting echo planar imaging distortions. Neuroimage.

[52] Culley D.J., Baxter M.G., Yukhananov R., Crosby G. (2004). Long-term impairment of acquisition of a spatial memory task following isoflurane-nitrous oxide anesthesia in rats. Anesthesiology.

[53] Jevtovic-Todorovic V. (2018). Exposure of Developing Brain to General Anesthesia. Anesthesiology.

[54] Rauh V.A., Perera F.P., Horton M.K., Whyatt R.M., Bansal R., Hao X., Liu J., Barr D.B., Slotkin T.A., Peterson B.S. (2012). Brain anomalies in children exposed prenatally to a common organophosphate pesticide. Proc. Natl. Acad. Sci. USA.

[55] Struyfs H., Van Hecke W., Veraart J., Sijbers J., Slaets S., De Belder M., Wuyts L., Peters B., Sleegers K., Robberecht C. (2015). Diffusion Kurtosis Imaging: A Possible MRI Biomarker for AD Diagnosis?. J. Alzheimers Dis..

